# A Novel Nomogram for Predicting Prognosis and Tailoring Local Therapy Decision for Ductal Carcinoma In Situ after Breast Conserving Surgery

**DOI:** 10.3390/jcm11175188

**Published:** 2022-09-01

**Authors:** Feifei Xu, Lu Cao, Cheng Xu, Gang Cai, Rong Cai, Weixiang Qi, Shubei Wang, Kunwei Shen, Weimin Chai, Jiayi Chen

**Affiliations:** 1 Department of Radiation Oncology, Ruijin Hospital, Shanghai Jiaotong University School of Medicine, Shanghai 20025, China; 2 Comprehensive Breast Health Center, Ruijin Hospital, Shanghai Jiaotong University School of Medicine, Shanghai 20025, China; 3 Department of Radiology, Ruijin Hospital, Shanghai Jiaotong University School of Medicine, Shanghai 20025, China

**Keywords:** ductal carcinoma in situ, whole breast irradiation, nomogram, mammographic microcalcification, neutrophil/lymphocyte ratio

## Abstract

**Purpose:** We sought to explore the role of nomogram-combined biomarkers, mammographic microcalcification and inflammatory hematologic markers in guiding local therapy decisions in ductal carcinoma in situ (DCIS) subgroups with different ipsilateral breast tumour recurrence (IBTR) risk. **Methods:** Between January 2009 and December 2018, consecutive patients with DCIS and breast conserving surgery (BCS) were enrolled and randomly assigned to a training cohort (*n* = 181) and internally validation cohort (*n* = 78). Multivariate analyses were performed to identify predictors of IBTR. Model performance was evaluated by the concordance index (C-index) and calibration plot. The time-to-event curves were calculated by the Kaplan–Meier methods and compared by the log-rank test. **Results:** In total, 259 patients were enrolled and 182 of them received whole breast irradiation (WBI). After a median follow-up of 51.02 months, 23 IBTR events occurred in the whole cohort. By multivariate analyses of training cohort, presence of microinvasion, Ki67 index >14%, mammographic-clustered fine linear microcalcifications and neutrophil/lymphocyte ratio before BCS (preop-NLR), >1.1 remained independent risk factors of IBTR to develop a nomogram. The C-indexes of the nomogram were 0.87 and 0.86 in the training and internal validation set, respectively. Calibration plots illustrated good agreement between the predictions and actual observations for 5-year IBTR. Cut-off values of nomogram point were identified as 53 and 115 points, which divided all patients into low-, intermediate- and high-risk groups. Significant differences in IBTR existed between low-, intermediate- and high-risk subgroups (*p* < 0.01). For the whole cohort and ER-positive tumours, the benefit of WBI was found only in the intermediate-risk subgroup, but not in those with low or high risk. Fourteen out of 23 IBTRs occurred outside the original quadrant and all occurred in the high-risk group. **Conclusions:** The novel nomogram demonstrated potential to separate the risk of IBTR and locations of IBTR. For the whole cohort and ER-positive tumours, the benefit of WBI was restricted to an intermediate-risk subgroup.

## 1. Introduction

Ductal carcinoma in situ (DCIS) of the breast is known as a precursor of invasive ductal carcinoma (IDC) with heterogeneous risk of recurrence [1]. Randomized trials have demonstrated that whole breast irradiation (WBI) could significantly decrease the risk of ipsilateral breast tumour recurrence (IBTR) in patients with DCIS after breast-conserving surgery (BCS) [2,3,4,5]. The standard use of endocrine therapy (ET) has further significantly decreased the risk of recurrence in patients with oestrogen receptor (ER) positive DCIS [4,6]. Given the excellent survival prognosis of DCIS tumours and the limited survival benefit from WBI, controversy persists regarding whether the active local treatment represents over-treatment for some individuals who will never develop invasive disease within their lifetime [7]. Thus, discussions surrounding the safe de-escalation of local treatment of DCIS have taken centre-stage to identify the subgroups at a low rate of IBTR after BCS.

University of Southern California/Van Nuys Prognostic Index (USC/VNPI) scoring systems and several other prognostic systems composed of clinical and pathological characteristics have been established to identify patients at a low risk of IBTR [8,9,10,11,12]. Updated results of RTOG 9804 trial demonstrated that WBI following BCS was associated with a significant improved local control (12-year rate of IBTR: 11.4% vs. 2.8%) even in patients defined as “good-risk” DCIS [13]. Based on traditional pathological and anatomical characteristics, there exists no unified definition to stratify DCIS patients as “low-risk” that can be spared from WBI after BCS. Additional biomarkers are needed to improve the risk stratification. It has been found that the presence of microinvasion with DCIS was associated with an increased risk of IBTR after BCS [14]. The mammographic-clustered fine linear microcalcification (MCFM) at diagnosis was also demonstrated as a poor predictor for increased IBTR [15,16]. In invasive breast cancers (IBC), the high neutrophil/lymphocyte ratio (NLR) was demonstrated to be highly predictive of locoregional failure among triple negative breast cancer (TNBC) patients receiving neoadjuvant chemotherapy [17]. In previous studies, the neutrophils were demonstrated to be associated with pro-tumour activities in vivo such as enhanced angiogenesis, which contribute to tumour cell proliferation and promote metastatic potential of the tumour cells [18]. Lymphocytes, on the other hand, have been implicated in having an important role in cancer immune surveillance, and are hypothesized to suppress tumour maturation [19]. An increased concentration of intratumoral CD8+ cytotoxic lymphocytes in breast cancer has been strongly associated with decreased recurrence, and higher survival outcomes [20]. It is, hence, biologically plausible that imbalances in the peripheral NLR may be the proxy of the on-going inflammatory process in the tumour microenvironment and provide an insight into underlying tumour progression and prognosis in individuals with breast cancer [18]. This seems to further suggest that the NLR have the potential to be predictive markers in breast cancer. However, the association between NLR and local recurrence in DCIS patients has not been well illustrated. Whether the incorporation of those novel markers will increase the power of prognostic system and tailor therapeutic strategy as well warrants further study.

In present study, we aimed to establish a novel nomogram based on information including pathological characteristics, biomarkers, mammography microcalcification features and NLR to better separate the IBTR risk and guide individualized local therapy decisions for patients with DCIS after BCS.

## 2. Materials and Methods

### 2.1. Study Cohort

Medical records of consecutive patients with pathologically confirmed DCIS, including DCIS with microinvasion, and having received BCS in a single institution between January 2009 and December 2018, were retrospectively reviewed. Patients with unknown and positive surgical margin, mixed ductal carcinoma in situ and lobular carcinoma-in situ, simultaneous contralateral breast cancer, pathological positive lymph nodes, prior or concurrent malignancy (except non-melanoma skin cancer) were excluded.

### 2.2. Pathological and Mammographic Examination

DCIS was classified as a neoplastic proliferation of epithelial cells confined to the mammary ductal-lobular system [21]. Microinvasion was identified as no more than 0.1 cm of the invasive component [22]. Oestrogen receptor (ER) status, Ki67 index and HER2 status was determined by immunohistochemistry staining. Nuclear staining >10% for ER was considered as positive ER status [23]. HER2 positivity was defined as an expression level intensity of 3+ [24]. High Ki67 index was characterised as more than 14% of tumour cell nuclei with positive immunostaining above background level [25]. All mammograms were independently reviewed by two senior radiologists and characterised by the Breast Imaging-Reporting and Data System [26]. Microcalcifications were analysed based on: morphology (punctate/amorphous, coarse heterogeneous, fine pleomorphic or fine linear/linear branching) and distribution (linear/segmental, regional/diffuse and clustered/grouped). All the patients received peripheral complete blood count (CBC) before BCS and the NLR before BCS (preop-NLR) were then collected. The value of preop-NLR to predict the 5-year IBTR was analysed using a time-dependent receiver operating characteristic (ROC) curve analysis and the optimal cut-off values were determined as 1.1 (showed in Supplemental Figure S1).

### 2.3. Adjuvant Radiotherapy

For patients treated with WBI, the dose of 50 Gy in 25 fractions was prescribed to the ipsilateral whole breast. The decision of the tumour bed boost was at the discretion of the radiation oncologist. WBI was delivered by forward-planning field-in-field photons’ intensity modulated radiation therapy using standard medial and lateral tangents. The tumour bed boost was given using reduced tangents or electron beams with dose prescription of 10 Gy in 5 fractions. The volume delineation and definition were determined according to the Radiation Therapy Oncology Group (RTOG) guidelines [27].

### 2.4. Outcomes’ Definitions

IBTR was defined as any pathologically confirmed recurrence of DCIS or invasive carcinoma in the ipsilateral breast. Elsewhere, the failure event (EFE) was defined as any pathologically confirmed recurrence of DCIS or invasive carcinoma in other quadrants of the ipsilateral breast (far removed from the primary, treated lesion). All recurrences at distant sites were recorded as metastases. The follow-up time was calculated from the date of surgery to the date of the first event or last confirmed date of breast cancer disease-free status.

### 2.5. Development and Validation of the Nomogram

The study population was randomly dichotomized into two groups: 70% in the training and 30% in the internal validation group, respectively. The training data set (*n* = 181) was used for initial nomogram development. Multivariate analyses were performed using the Cox regression analysis with a forward stepwise selection. A nomogram for separating the IBTR risk was developed based on the independent risk factors identified in multivariate analysis. Calibration curves were plotted to assess the agreement between the actual rate and the predicted probabilities of IBTR, which were assessed by calibration plot with 1000 bootstrap resampling. The predictive accuracy was evaluated by Harrell’s concordance index (c-index), which ranges from 0.5 (random chance) to 1 (perfect prediction). The nomogram was further validated using the internal validation group (*n* = 78). Finally, different IBTR risk groups were developed based on the novel nomogram.

### 2.6. Statistical Analysis

The software package SPSS 25.0 (IBM corporation, New York, NY, USA) and R software was used for analysis. Continuous variables were summarized by median and range, and categorical variables were summarized by frequency and proportion. The Pearson’s Chi-square test (Fisher’s exact test when necessary) was used to compare the distribution of clinicopathological features between groups. The time-to-event curves were calculated by the Kaplan–Meier methods and compared by the log-rank test. Multivariate analyses were performed using a Cox regression analysis with forward stepwise selection. Only the variables that show evidence of association (*p* ≤ 0.05) in the univariate analysis were tested in the multivariate analysis. Adjusted hazard ratios with 95% confidence intervals (CIs) were reported. All statistical tests were two-sided and *p* ≤ 0.05 was considered significant.

## 3. Results

### 3.1. Patient and Treatment Characteristics

In total, 259 patients were enrolled in this analysis. The median age was 49 years (range, 25–92) and median maximum tumour size was 1.5 cm (range, 0.1–5.0). There were 48 (18.5%) patients with presence of microinvasion. Among 184 patients with ER-positive tumours, 82.1% received endocrine therapy. Compared with those treated with BCS only, patients who received WBI were more likely to be premenopausal, high grade and high preop-NLR (*p* = 0.02, *p* = 0.04 and *p* = 0.01, respectively). There was no significant difference in other clinicopathologic characteristics between the BCS + WBI and BCS only group. Out of the 182 patients treated with WBI, 71.4% received a sequential tumour bed boost. The whole patient and treatment characteristics according to delivery of WBI are detailed in Table 1.

### 3.2. IBTR Events and Survival Outcomes

With a median follow-up of 51.02 months (range, 0.53–136.87), 23 IBTR events occurred. In whole cohort, the 5-year rate of IBTR was 9.2%, WBI reduced the 5-year rate of IBTR from 12.2% to 8.2% (*p* = 0.14). With respect to invasive-IBTR, 10 invasive-IBTR events occurred with 9 in the BCS + WBI group and 1 in the BCS only group (*p* = 0.25). For patients with ER-positive tumours, the 5-year rate of IBTR was 3.4% and 9.1% in the BCS + WBI group and BCS only group, respectively (*p* = 0.06). In the whole cohort, two patients developed recurrence in the ipsilateral axillary and supraclavicular lymph nodes, four patients developed bone and/or brain metastases, and four patients died, while only one patient died of breast cancer.

### 3.3. Relationship between NLR and TNBC

In our study, there were 37 patients with the TNBC tumour, in which there were 13 patients with high preop-NLR. With respect to high preop-NLR, there was no significant difference between the TNBC and ER-positive and HER2-positive group (64.9% vs. 63.6% vs. 71.4%, *p* = 0.60) in our DCIS cohort. In the TNBC cohort, the 5-year rate of IBTR was extremely higher in high preop-NLR group than the low preop-NLR group (0.0% vs. 24.5%, *p* = 0.04). However, it was not associated with a significant increase in 5-year IBTR between the low and high preop-NLR group with 0.0% vs. 4.3% in the ER-positive group (*p* = 0.06) and 9.1% vs. 33.3% (*p* = 0.07) in the HER2-positive subgroup, respectively. In invasive breast cancer (IBC), previous studies have well demonstrated that higher pre-treatment NLR was independently correlated with poor DFS and OS in TNBC patients [28,29]. However, data on the association between NLR and TNBC in DCIS cohort was much less documented. Our results revealed that, similar to the results from the IBC cohort, the higher preop-NLR was also related to the poor prognosis in TNBC DCIS population.

### 3.4. Nomogram Development

We randomly divided the whole patients into a training cohort and an internal validation cohort (7:3). 

**Figure 1:**
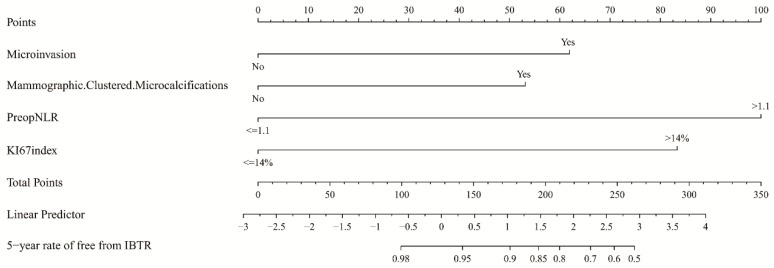
Nomogram for predicting 5-year IBTR in the training cohort. Abbreviations: IBTR = ipsilateral breast tumour recurrence; NLR = neutrophil/lymphocyte ratio.

The randomization processes were carried out by using the sample-split function of the “caTools” package in R software, and we set the Split-Ratio as 0.7, which could randomly divide the whole patients into a training and internal validation cohort (7:3). In total, 181 and 78 patients were enrolled in the training and internal validation group, respectively. For the training group, the median age was 49 years (range, 26–92), and the median maximum tumour size was 1.3 cm (range, 0.1–4.0). There were 17.7% patients with a presence of microinvasion, 69.1% with the high preop-NLR and 83% of ER-positive patients who received endocrine therapy. For the internal validation group, the median age was 48 years (range, 25–80) and the median maximum tumour size was 1.5 cm (range, 0.2–5.0). There were 16.7% patients with the presence of microinvasion, 56.4% with the high preop-NLR and 79.6% of ER-positive patients who received endocrine therapy. There were 127 and 55 patients treated with WBI in the training and internal validation group, respectively. No variables were significantly different between these two cohorts, which are detailed in Supplemental Table S1. The patients of the training group were eventually reviewed to develop the nomogram. The univariable analysis for IBTR demonstrated that the tumour size (≤2.5 cm vs. >2.5 cm), nuclear grade (Low-Intermediate vs. High), microinvasion (Yes vs. No), ER status (positive vs. negative), HER2 status (positive vs. negative), Ki67 index (≤14% vs. >14%), mammographic-clustered fine linear microcalcifications (Yes vs. No) and preop-NLR (≤1.1 vs. >1.1) were associated with a high risk of IBTR (*p* = 0.04, *p* = 0.04, *p* < 0.01, *p* < 0.01, *p* < 0.01, *p* < 0.01, *p* = 0.01 and *p* = 0.01, respectively). In the multivariable analysis, the presence of microinvasion (HR = 3.37, 95% CI 1.11–10.20, *p* = 0.03), Ki67 index >14% (HR = 5.63, 95% CI 1.47–21.55, *p* = 0.01), presence of mammographic-clustered fine linear microcalcifications (HR = 3.32, 95% CI 1.12–9.85, *p* = 0.03) and preop-NLR >1.1 (HR = 7.87, 95% CI 0.99–62.63, *p* = 0.05) remained independent risk factors for IBTR (shown in Table 2). Variables that were statistically significant in the multivariable analysis were used to construct the nomogram. For each patient, the total points were calculated by adding up the score of each variable to predict the probability of rate of IBTR (Figure 1).

### 3.5. Performance of the Nomogram and Internal Validation

**Figure 2:**
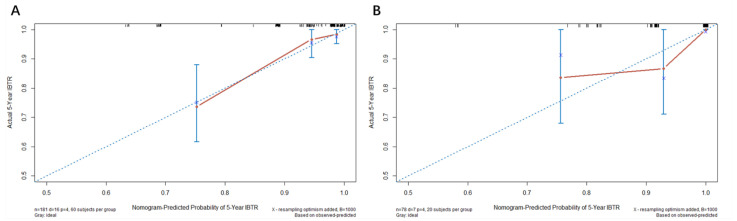
Calibration curves for 5-year IBTR in the training (**A**) and internal validation cohort (**B**). Abbreviations: IBTR = ipsilateral breast tumour recurrence.

The C-index of nomogram in the training data was 0.87, and the calibration plot indicated a good concordance between the predicted and observed 5-year IBTR probabilities (as shown in Figure 2A). In the internal validation data of 78 patients, the C-index of the nomogram was 0.86 and the calibration curve also revealed good concordance (Figure 2B). The prognostic model-predicted IBTR with 5-year AUC was 0.85 with a 95% confidence interval (CI) ranging from 0.74 to 0.95 in whole cohort, which indicated a strong model, as shown in Supplemental Figure S2.

### 3.6. IBTR Risk Group

**Figure 3:**
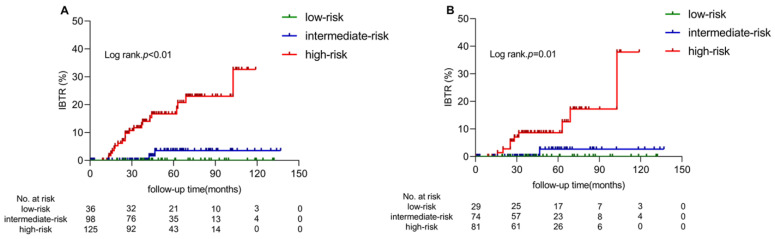
Cumulative incidence of IBTR according to IBTR risk group in all patients (**A**) and patients with ER-positive tumours (**B**). Abbreviations: IBTR = ipsilateral breast tumour recurrence.

With the optimal cut-off values (53 and 115) selected by the X-tile, the whole patients were categorized as low-risk (total points ≤ 53), moderate-risk (50 < total points ≤ 115) and high-risk (total points > 115) groups. The number of patients in the low-risk, intermediate-risk and high-risk were 36, 98 and 125, respectively. The average NLR in high, intermediate and low-risk group patients was 1.80, 1.60 and 1.15, respectively. Five-year IBTR rates were 0.0%, 3.5% and 16.7% in the low-, intermediate- and high-risk subgroups, respectively (*p* < 0.01, as shown in Figure 3A). Among 184 patients with an ER-positive tumour, the IBTR risk groups also demonstrated significance in dividing the IBTR risk (*p* = 0.01, as shown in Figure 3B).

### 3.7. Benefit from WBI According to IBTR Risk Groups

In the whole cohort, WBI was associated with a significant reduction of 5-year IBTR from 13.5% to 0.0% in patients with intermediate-risk (*p* = 0.02), while the 5-year rate of IBTR between the BCS only and BCS + WBI group was 0% vs. 0% in the low-risk subgroup and 16.5% vs. 16.7% (*p* = 0.34) in the high-risk subgroup, respectively.

In patients with ER-positive tumours, similar to the results from the whole cohort, WBI only decreased the 5-year rate of IBTR from 11.1% to 0% in the intermediate-risk subgroup (*p* = 0.08), with no significant benefit in IBTR in the low-risk subgroup (5-year rate of 0% vs. 0%) and high-risk subgroup (5-year rate of 10.5% vs. 7.8%, *p* = 0.37).

We further explore the prognosis separately in the BCS + WBI and BCS group by utilizing the nomogram based on the training group of whole cohorts. For the BCS + WBI group, the 5-year rates of IBTR were significantly different in different risk subgroups with 0.0%, 0.0% and 16.7% in the low-, intermediate- and high-risk subgroups, respectively (*p* < 0.01). For the BCS-only group, the 5-year rates of IBTR were 0.0%, 13.5% and 16.5% in the low-, intermediate- and high-risk subgroups, respectively (*p* = 0.10).

### 3.8. Pattern of IBTR with Regard to Risk Groups

Fourteen out of 23 IBTRs occurred outside of the original quadrant (elsewhere failure event, EFE) and the 5-year rate of EFE was 4.8%. Eight out of 14 IBTRs in the BCS + WBI group and 6 out of 9 in the BCS-only group were EFEs (*p* = 0.11). Classified by the IBTR risk groups, all 14 EFEs occurred in the high-risk group (*p* < 0.01).

## 4. Discussion

The role of radiotherapy in the treatment of DCIS after BCS has been an issue of debate for decades. To our knowledge, this is the first study to establish a nomogram combining radiological and inflammatory hematologic characteristics with biomarkers aimed to improve individualized local treatment decision. The novel nomogram in our study was composed of microinvasion, Ki67 index, mammographic-clustered fine linear microcalcifications and preop-NLR, which not only separated patients into three distinct risk groups of IBTR, but also helped to define the population that might most benefit from WBI. The benefit of WBI was most observed in the intermediate-risk group. However, all EFEs occurred in the high-risk group, which indicated that BCS should be recommended with caution in population with two or more of the aforementioned risk factors.

According to the nomogram established in our study, the risk of IBTR varied in three different risk subgroups, from a 5-year rate of 0.0% in the low-risk group to 16.7% in the high-risk group. DCIS is a heterogeneous disease with a wide range of recurrence risk with 50% invasive recurrence [5]. A number of risk factors for invasive recurrence in DCIS patients have been reported, including clinicopathologic and radiological features, molecular subtypes, multigene signatures and tumour-immune microenvironments. Toss et al. indicated that the ER-negative status was associated with a statistically significantly increased recurrence rate of invasive carcinomas in DCIS patients [30]. Williams et al. also revealed invasive recurrence rates that differed between phenotypes. A total of 1.3% of Luminal A cases had an invasive recurrence, compared to 16.1% of the Luminal B, 29.5% of the HER2 type and 23.1% of TNBC at 10 years follow-up [31]. Poulakaki et al. highlighted the detrimental association of high Ki-67 index with a higher rate of invasive recurrence increased by 66% [32]. In a case-cohort study within a randomized trial of DCIS treated by breast conserving surgery (SweDCIS), Holmberg et al. have reported a significant association between fine linear microcalcifications and increased invasive recurrence [15]. A recent study revealed that the 5-year rate of invasive recurrence was significantly higher in the dense density of tumour-infiltrating lymphocytes (TILs) group compared with the sparse TILs group (12.6% vs. 0.0%, *p* < 0.01) [33]. This nomogram was developed based on four prognostic factors for IBTR in DCIS that included the Ki67 index, microinvasion, mammographic-clustered fine linear microcalcifications and preop-NLR. Lazzeroni et al. identified the Ki67 index (>14%) as an independent prognostic factor for high IBTR in a cohort of 1171 consecutive patients with DCIS [34]. The population study by Lalani N et al., which enrolled women with DCIS treated by BCS (2721 with pure DCIS and 267 with microinvasion) has validated that the 15-year rate of IBTR for DCIS with microinvasion versus pure DCIS was 34% versus 25% (*p* = 0.03) [35]. One of the important findings in our study is the power of IBTR risk stratification of mammographic-clustered fine linear microcalcifications. The implement of mammography screening effectively helped to detect early DCIS with no clinical palpable mass [36]. With mammography, 85% of DCIS patients could be diagnosed at early onset and 75–90% of these cases appeared as microcalcifications at mammography [37]. Consistent with our results, the study with the largest sample to date about relationship between microcalcifications and IBTR risk published by Rauch GM et al. proved that fine linear microcalcification was an independent prognostic indicator in 987 patients treated with BCS, which was associated with a 5.13-fold increase in the risk of IBTR [26].

It is also worth mentioning that this is the first study to assess the prognostic and predictive significance of NLR in DCIS patients who received BCS. Inflammatory hematologic parameters, especially high NLR, have been validated to be associated with higher pCR and to benefit from radiotherapy for IBC [38,39]. However, data on the prognostic and predictive significance of NLR for DCIS was much less demonstrated. A previous study suggested that high NLR could be associated with the immunosuppression of the tumour-immune microenvironment [40]. In the current study, we found that a relatively high preop-NLR in DCIS was associated with an increased risk of tumour recurrence. One postulation was that high NLR was associated with an abnormal host-immune surveillance status, which might contribute to tumour proliferation, invasion and metastasis [39]. In this context, we suggest that the mammographic feature and inflammatory hematologic parameters could be integrated in evaluating the individualized risk of IBTR of DCIS and to tailor the local treatment accordingly.

Several prognostic models have been reported for predicting IBTR based on clinical and pathologic characteristics prior to our study. By quantifying four measurable prognostic factors of tumour size, margin width, nuclear grade and age, the USC/VNPI scoring system divided patients into three groups with different risk of IBTR, as determined by scores of 4–6, 7–9 and 10–12 [12]. The Oncotype DX DCIS Score and DCISionRT test also demonstrated the potential to quantify individualized IBTR risk in patients with DCIS and BCS alone [41,42]. Nevertheless, the existing prognostic scoring systems all focused on pure DCIS and seldom integrated information from NLR and mammographic features. We tried to evaluate the predictive value of USC/VNPI score system in our cohort and found that there was no patient that met the criteria of a high-risk score of USC/VNPI, which is 10–12. Nevertheless, in our defined high-risk group, the 5-year rate of IBTR remained as high as 16.7% even with the addition of WBI. Moreover, all of the 14 EFEs occurred in the high-risk group, which is by far the first study to describe the pattern of IBTR with regard to different risk groups. Thus, there exists a need for a more sophisticated nomogram so as to further separate the risk of IBTR and to redefine a subset that BCS should probably not to be recommended to even with the addition of WBI because of high IBTR, especially high EFEs, while this population otherwise would have been defined as candidates for BCS according to the USC/VNPI scoring system. Adjuvant endocrine therapy has further kept the risk of IBTR in ER-positive DCIS to a considerably low level [4,6]. The risk stratification system developed by our nomogram persists its power in the ER-positive tumours, in which the majority of patients had endocrine therapy.

Although WBI was proved to significantly reduce the rate of local recurrence by 50% in the DCIS population, the survival benefit from WBI was limited. With the excellent survival prognosis of DCIS, the balance between the treatment benefits and adverse effects from radiation became the most concerning for doctors and patients. The adjuvant breast RT was associated with short- and long-term adverse effects, such as cosmetic skin effects, radiation pneumonitis and heart problems [43,44]. In addition, unnecessary irradiation was associated with substantial health care resource demands [45,46]. A retrospective review, which identified patients who underwent lumpectomy for breast cancer in Massachusetts General Hospital, has revealed that no-WBI represented cost savings of US$ 13.36 million per 1000 patients than receiving WBI [45]. Moreover, the outbreak of the COVID-19 pandemic has now impacted the radiotherapy strategy for breast cancer patients. A multi-centre cross-section survey performed by the Ruijin Hospital revealed that 54.3% of patients changed the radiation strategy during the pandemic and the interruption during radiation significantly increased the fear of cancer recurrence of patients [47]. Individualized radiotherapy for DCIS after breast conserving surgery is of importance to minimize both over treatment and under adverse effects of treatment and improve psychological status. How to define a subgroup of DCIS that can be safely spared from WBI remains controversial. Based on our nomogram, the benefit of additional WBI was limited to patients in the intermediate-risk subgroup with an absolute reduction of a 5-year rate of 13.5%. Patients with a low-risk score might be candidates for BCS only. Consistently with our finding, in a large prospective trial of 1564 patients with DCIS and BCS, Rakovitch et al. also found that significant reduction in the IBTR risk following WBI only existed in patients with intermediate Oncotype DX DCIS score (39 to 56) but not in those with a low (<39) and high DCIS score (≥54) [48]. In the RTOG 9804 trial, the “good-risk” was defined as detected by the mammogram, as tumours of ≤2.5 cm, low or intermediate nuclear grade and with margins’ width of ≥3 mm. In the updated results after a median follow-up of 12 years, the WBI continuously reduced the rate of IBTR significantly from 11.4% to 2.8% [13]. Compared with RTOG 9804, the IBTR rate was quite low in the low-risk group of our study. Except for a small sample size and relative short follow-up, a more restricted definition of low-risk group was established by our nomogram. A total of 29 out of 36 patients with low-risk were ER-positive and 86.2% of them received endocrine therapy in our study, while only 69% of the whole cohort intended to receive tamoxifen in the RTOG 9804 trial, which also partly explains the very low IBTR observed in our study. Continuous efforts are being made to explore the feasibility of omitting WBI in low-risk invasive cancer, while the definition integrated traditional and clinical pathological characteristics, biomarkers and multi-gene assays. In DCIS patients, the ongoing ROMANCE trial from France also tried to integrate the value of biomarkers, which selected characteristics of ≥50 years, with a tumour size of ≤2.5 cm, low-intermediate nuclear grade, margins’ width of ≥2 mm and luminal-A subtype as “low-risk” criteria [49]. We would speculate that the risk of IBTR and the benefit of WBI might be better stratified.

As a retrospective study, our study does have several inherent limitations. The median follow-up of 51.02 months is relatively short and might underestimate the actual risk of IBTR. The limited whole sample size and small number of IBTR events could also be a potential source of bias. Prospective studies with a large sample and long-term follow up are essentially to verify the prognostic and predictive value of the nomogram found in our study.

## 5. Conclusions

The novel nomogram composed of Ki67 index, microinvasion, mammographic-clustered fine linear microcalcifications and preop-NLR could separate the risk of IBTR and help to tailor local treatment decisions. For the whole cohort of DCIS and subgroup of ER-positive tumours, the benefit of WBI were most observed in patients in the intermediate-risk group. Patients in the high-risk group not only showed high IBTR, but also a significantly higher risk of failure outside primary quadrants.

## Figures and Tables

**Table 1:** Patient and treatment characteristics.

Characteristics	Whole Cohort		BCS Only		BCS + WBI		*p* Value
	*n* = 259	%	*n*= 77	%	*n* = 182	%	
Age (years)							
Median (range)	49 (25–92)		52 (25–92)		48(26–80)		
<40	39	15.1	8	10.4	31	17	0.19
≥40	220	84.9	69	89.6	151	83	
Menopausal status							0.02
Premenopausal	137	52.9	32	41.6	105	57.7	
Postmenopausal	122	47.1	45	58.4	77	42.3	
Tumour size (cm)							
Median (range)	1.5 (0.1–5.0)		1.0 (0.15–3.5)		1.5 (0.1–5.0)		
≤2.5	235	90.7	72	93.5	163	89.6	0.36
>2.5	24	9.3	5	6.5	19	10.4	
Nuclear grade							
Low-Intermediate	178	68.7	60	77.9	118	64.8	0.04
High	81	31.3	17	22.1	64	35.2	
Comedo-necrosis							
Yes	59	22.8	16	20.8	43	23.6	0.75
No	220	77.2	61	79.2	139	76.4	
Microinvasion							
Yes	48	18.5	9	11.7	39	21.4	0.08
No	211	81.5	68	88.3	143	78.6	
ER status							
Positive	184	71.0	55	71.4	129	70.9	1.00
Negative	75	29.0	22	28.6	53	29.1	
Ki67 index							
≤14%	166	64.1	54	70.1	112	61.5	0.21
>14%	93	35.9	23	29.9	70	38.5	
HER2 status							
Positive	54	20.8	14	18.2	40	22	0.62
Negative	205	79.2	63	81.8	142	78	
Mammographic-clustered microcalcifications							
Yes	86	33.2	25	32.5	61	33.5	1.00
No	173	66.8	52	67.5	121	66.5	
Preop-NLR							
≤1.1	73	28.2	12	15.6	61	33.5	0.01
>1.1	186	71.8	65	84.4	121	66.5	
Axillary surgery							
SLNB	117	45.2	31	40.3	86	47.3	0.56
ALND	17	6.6	5	6.5	12	6.6	
No surgery	125	48.3	41	53.2	84	46.2	
Endocrine therapy in ER positive (*n* = 184)							0.21
Yes	151	82.1	42	76.4	109	84.5	
No	33	17.9	13	23.6	20	15.5	
Target therapy							
Yes	1	0.4	0	0	1	0.5	0.52
No	258	99.6	77	100	181	99.5	
Chemotherapy							
Yes	6	2.3	1	1.3	5	2.7	0.48
No	253	97.7	76	98.7	177	97.3	

Abbreviations: BCS = breat canserving surgery; WBI = whole breast irradiatin; ER = estrogen receptor; HER2 = human epidermal growth factor receptor 2; NLR = neutrophil/lymphocyte ratio; SLNB = sentinel lymph node biopsy; ALND = axillary lymph node dissection.

**Table 2:** The univariate and multivariable analyses for IBTR in training group. Abbreviations: IBTR = ipsilateral breast tumour recurrence; ER = estrogen receptor; HER2 = human epidermal growth factor receptor 2; NLR = neutrophil/lymphocyte ratio; SLNB = sentinel lymph node biopsy; ALND = axillary lymph node dissection.

Characteristics	IBTR					
	Univariate Analyses			Multivariable Analyses		
	*n* of IBTR	5-Year Rate	*p* Value	HR	95% CI	*p* Value
Age (years)			0.77			
<40	3	14.5				
≥40	13	7.4				
Menopausal status			0.53			
Premenopausal	10	12.3				
Postmenopausal	6	5.9				
Tumour size (cm)						
≤2.5	12	8.1	0.04	1		
>2.5	4	19.8		2.04	0.58–7.13	0.27
Nuclear grade			0.04			
Low-Intermediate	8	5.4		1		
High	8	19.2		0.74	0.25–2.15	0.57
Comedo-necrosis			0.17			
Yes	6	17.1				
No	10	7.2				
Microinvasion			<0.01			
Yes	7	24.7		1		
No	9	6.1		3.37	1.11–10.20	0.03
ER status			<0.01			
Positive	7	5.1		1		
Negative	9	20.6		0.66	0.18–2.43	0.53
Ki67 index			<0.01			
≤14%	3	1.8		1		
>14%	13	24.0		5.63	1.47–21.55	0.01
HER2 status			<0.01			
Positive	8	29.1		1		
Negative	8	4.5		1.17	0.34–3.92	0.80
Mammographic-clustered microcalcifications			0.01			
Yes	11	16.3		1		
No	5	5.4		3.32	1.12–9.85	0.03
Preop-NLR			0.01			
≤1.1	1	2.2		1		
>1.1	15	12.9		7.87	0.99–62.63	0.05
Axillary surgery			0.41			
SLNB	9	13.7				
ALND	1	16.7				
No surgery	6	5.2				

## Supplementary Materials

The following supporting information can be downloaded at: https://www.mdpi.com/article/10.3390/jcm11175188/s1, Figure S1: The time-dependent receiver operating characteristic (ROC) curve analysis of the ability of NLR to predict the 5-year IBTR (AUC = 0.54); Figure S2: ROC analysis of nomogram for predicting 5-year IBTR; Table S1: Patient and Treatment Characteristics between the training group and the internal validation group.
