# Supplementary material for: A Novel Nomogram for Predicting Prognosis and Tailoring Local Therapy Decision for Ductal Carcinoma In Situ after Breast Conserving Surgery
Source: J Clin Med. 2022 Sep 1;11(17):5188. doi: 10.3390/jcm11175188 (PMC9456583; doi:10.3390/jcm11175188)
Supplement: Supplementary file 1 [file jcm-11-05188-s001.zip › jcm-1802863-supplementary.pdf]

Supplemental Figure S1: The time-dependent receiver operating characteristic (ROC) curve analysis of the ability of NLR to predict the 5-year IBTR (AUC=0.54).

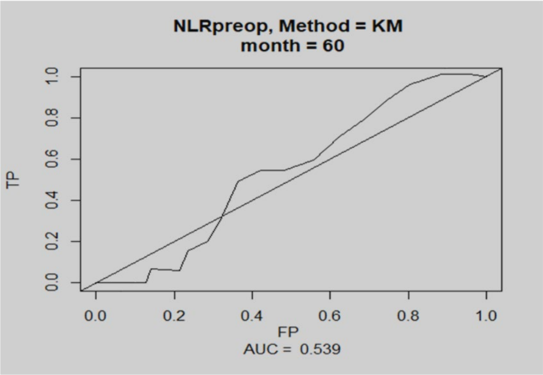

Supplemental Figure S2: ROC analysis of nomogram for predicting 5-year IBTR.

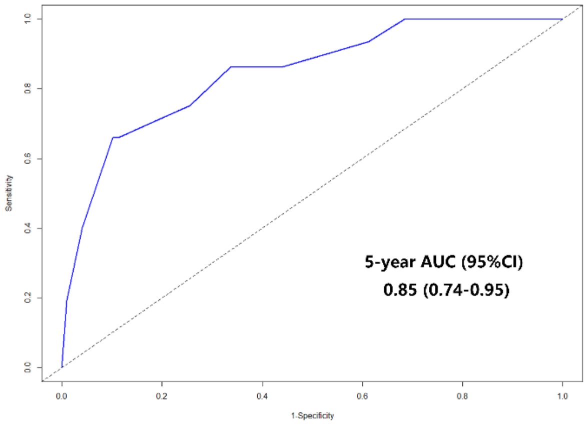

Supplemental Table S1: Patient and Treatment Characteristics between the training group and the internal validation group.

Characteristics	Training group		Testing group		P value
	N=181	%	N=78	%	
**Age (years)**					
Median (range)	49 (26-92)		48 (25-80)		0.73
<40	25	13.8	14	17.9	0.45
≥40	156	86.2	64	82.1	
**Menopausal status**					0.79
Premenopausal	97	53.6	40	51.3	
Postmenopausal	84	46.4	38	48.7	
**Tumor size (cm)**					
Median (range)	1.3 (0.1-4.0)		1.5 (0.2-5.0)		0.40
≤2.5	165	91.2	69	88.5	0.50

Characteristics	Training group		Testing group		P value
	N=181	%	N=78	%	
>2.5	16	8.8	9	11.5	
**Nuclear Grade**					0.15
Low-Intermediate	129	71.3	48	61.5	
High	52	28.7	30	38.5	
**Comedo-necrosis**					0.20
Yes	37	20.4	22	28.2	
No	144	79.6	56	71.8	
**Microinvasion**					1.00
Yes	32	17.7	13	16.7	
No	149	82.3	65	83.3	
**ER status**					0.07
Positive	135	74.6	49	62.8	
Negative	46	25.4	29	37.2	
**Ki67 index**					0.40
≤14%	119	65.7	47	60.3	
>14%	62	34.3	31	39.7	
**HER2 status**					0.13
Positive	33	18.2	21	26.9	
Negative	148	81.8	57	73.1	
**Mammographic Clustered Microcalcifications**					0.20
Yes	65	35.9	21	26.9	
No	116	64.1	57	73.1	
**Preop-NLR**					0.06
≤1.1	56	30.9	34	43.6	
>1.1	125	69.1	44	56.4	
**Axillary surgery**					0.01
SLNB	84	46.4	33	42.3	
ALND	6	3.3	11	14.1	

Characteristics	Training group		Testing group		P value
	N=181	%	N=78	%	
No surgery	91	50.3	34	43.6	
**Endocrine therapy in ER positive (n=184)**					0.67
Yes	112	83.0	39	79.6	
No	23	17.0	10	20.4	
**Target therapy**					1.00
Yes	1	0.6	0	0	
No	180	99.4	78	100	
**Chemotherapy**					1.00
Yes	4	2.2	2	2.6	
No	177	97.8	76	97.4	
